# Supplementary material for: Socioeconomic inequalities in childhood and adolescent obesity in Australia: The role of behavioral and biological factors
Source: PLoS One. 2025 Apr 16;20(4):e0321861. doi: 10.1371/journal.pone.0321861 (PMC12002548; doi:10.1371/journal.pone.0321861)
Supplement: S1 Appendix — (DOCX) [file pone.0321861.s001.docx]

**S1: Contribution of key variables by concentration index (B-Cohort)**

| **Variables under study** | **Wave 2(n=4605)** | **Wave 3(n=4386)** | **Wave 4 (n=4242)** | **Wave 5 (n=4085)** | **Wave 6(n=3764)** | **Wave 7 (n=3381)** | **Pooled** |
| --- | --- | --- | --- | --- | --- | --- | --- |
| Behavioral factors | 0.0000 | 0.0001 | 0.0003 | 0.0002 | 0.0008 | 0.0000 | -0.0002 |
| Biological factors | -0.0007 | -0.0013 | -0.0017 | -0.0026 | -0.0031 | -0.0034 | -0.0003 |
| Household income | -0.0009 | -0.0018 | -0.0028 | -0.0048 | -0.0038 | -0.0055 | -0.0007 |
| Mother education | -0.0004 | 0.0000 | -0.0008 | -0.0010 | -0.0014 | -0.0009 | -0.0001 |
| Father education | -0.0002 | -0.0003 | -0.0003 | -0.0010 | -0.0009 | -0.0013 | -0.0002 |
| Mother employment | 0.0006 | 0.0001 | 0.0000 | 0.0014 | 0.0012 | 0.0019 | 0.0001 |
| Father employment | -0.0002 | 0.0003 | 0.0000 | -0.0003 | 0.0005 | 0.0004 | 0.0000 |
| Sociodemographic (age, gender, place of residence) | 0.0000 | 0.0000 | 0.0000 | 0.0000 | 0.0000 | 0.0001 | 0.0001 |
| CI of BMI | -0.002 | -0.0028 | -0.0054 | -0.008 | -0.0069 | -0.009 | -0.001 |
| Total estimated contribution | -0.002 | -0.0027 | -0.0054 | -0.008 | -0.0068 | -0.0087 | -0.001 |
