# Supplementary material for: Socioeconomic inequalities in childhood and adolescent obesity in Australia: The role of behavioral and biological factors
Source: PLoS One. 2025 Apr 16;20(4):e0321861. doi: 10.1371/journal.pone.0321861 (PMC12002548; doi:10.1371/journal.pone.0321861)
Supplement: S2 Appendix — (DOCX) [file pone.0321861.s002.docx]

**Contribution of key variables by concentration index (K-Cohort)**

| **Variables** | Wave1 **(n=4982)** | Wave2 **(n=4464)** | Wave3 **(n=4331)** | Wave4 **(n=4169** | Wave5 **(n=3958** | Wave6 **(n=3537** | Pooled |
| --- | --- | --- | --- | --- | --- | --- | --- |
| Behaviors factors | 0.000 | 0.0002 | 0.0005 | 0.0000 | 0.0007 | 0.0002 | -0.0001 |
| Biological factors | -0.0002 | -0.001 | -0.002 | -0.002 | -0.003 | -0.003 | -0.0003 |
| Household income | -0.001 | -0.001 | -0.004 | -0.001 | -0.003 | -0.003 | -0.0005 |
| Mother education | 0.0006 | 0.0000 | -0.0002 | -0.0011 | -0.0013 | -0.0001 | 0.0000 |
| Father education | -0.001 | -0.001 | -0.002 | -0.002 | -0.002 | -0.001 | -0.0002 |
| Mother employment | 0.0004 | 0.0008 | 0.0015 | 0.0007 | 0.0010 | 0.0000 | 0.0001 |
| Father employment | -0.0009 | -0.0013 | -0.0019 | -0.0020 | -0.0021 | -0.0015 | 0.0000 |
| Sociodemographic (age, gender, place of residence) | 0.0000 | -0.0001 | 0.0000 | 0.0001 | -0.0001 | 0.0000 | 0.0005 |
| CI of BMI | -0.0016 | -0.003 | -0.005 | -0.005 | -0.009 | -0.008 | -0.0006 |
| Total estimated contribution | -0.0015 | -0.003 | -0.005 | -0.005 | -0.009 | -0.008 | -0.00056 |
