# Supplementary material for: Socioeconomic inequalities in childhood and adolescent obesity in Australia: The role of behavioral and biological factors
Source: PLoS One. 2025 Apr 16;20(4):e0321861. doi: 10.1371/journal.pone.0321861 (PMC12002548; doi:10.1371/journal.pone.0321861)
Supplement: S3 Appendix — (DOCX) [file pone.0321861.s003.docx]

**Descriptive statistics for B cohort and K cohort**

| **Variables** | **B-cohort** | | **K- cohort** | |
| --- | --- | --- | --- | --- |
|  | **Wave 2** | **Wave 7** | **Wave 1** | **Wave 6** |
| **Dependent variable** | | | | |
| Body Mass Index (BMI) of children | 16.3 | 19.83 | 15.85 | 21.41 |
| **Independent variables** | | | | |
| **Consumption of fruit and Vegetables (e.g., fresh fruits, cooked vegetables, and raw vegetables) in the last 24 hours** | | | | |
| One or more than one time in a day | 0.88 | 0.847 | 0.911 | 0.858 |
| Not at all | 0.12 | 0.153 | 0.089 | 0.142 |
| **Consumption of fatty foods (e.g., French fries, savory food, biscuits, and pie) in the last 24 hours (what are the fatty goods)** | | | | |
| One or more than one time in a day | 0.966 | 0.909 | 0.956 | 0.94 |
| Not at all | 0.34 | 0.091 | 0.044 | 0.06 |
| **Drinking sugary beverages (e.g., fruits juice, soft drink/cordial)** | | | | |
| One or more than one time in a day | 0.697 | 0.581 | 0.802 | 0.714 |
| Not at all | 0.303 | 0.419 | 0.198 | 0.286 |
| **Activity during free time** | | | | |
| Riding bike/dancing/walking | 0.801 | 0.551 | 0.742 | 0.535 |
| Screening time | 0.199 | 0.449 | 0.248 | 0.465 |
| ***Outdoor activities*** |  |  |  |  |
| Gone for swimming with your parents in the past month? | 0.922 | 0.628 | 0.934 | 0.372 |
| Involved in a school event or community event with parents in the past month | 0.42 | 0.483 | 0.664 | 0.43 |
| Gone for watching a sports event with parents in the past month? | 0.383 | 0.757 | 0.605 | 0.724 |
| Gone for attending a religious service with parents in the past month? | 0.268 | 0.295 | 0.356 | 0.262 |
| Visited a library with parents in the past month? | 0.347 | 0.266 | 0.52 | 0.186 |
| **Biological factors** | | | | |
| Mother BMI | 24.13 | 26.9 | 24.89 | 26.81 |
| Father BMI | 26.36 | 27.41 | 26.19 | 27.74 |
| **Household Income** | | | | |
| Lowest income (500 AUD or less per week) | 0.52 | 0.309 | 0.686 | 0.33 |
| lowest to medium (501 to 1000 AUD per week) | 0.379 | 0.379 | 0.26 | 0.364 |
| Medium to highest (1001 to 1500 AUD per week) | 0.085 | 0.266 | 0.048 | 0.261 |
| Highest (more than 1501 AUD per week) | 0.016 | 0.046 | 0.005 | 0.041 |
| **Mother’s education** | | | | |
| Postgraduation | 0.076 | 0.102 | 0.061 | 0.091 |
| Undergraduate | 0.263 | 0.291 | 0.23 | 0.258 |
| Certificate/Diploma | 0.643 | 0.589 | 0.695 | 0.634 |
| Year 12 or below | 0.018 | 0.018 | 0.014 | 0.017 |
| **Father’s education** | | | | |
| Postgraduation | 0.073 | 0.089 | 0.071 | 0.089 |
| Undergraduate | 0.192 | 0.202 | 0.183 | 0.18 |
| Certificate/Diploma | 0.703 | 0.678 | 0.726 | 0.71 |
| Year 12 or below | 0.032 | 0.031 | 0.02 | 0.021 |
| **Mother’s employment** | | | | |
| Full-time employed | 0.42 | 0.384 | 0.214 | 0.501 |
| Part-time Employed | 0.172 | 0.44 | 0.364 | 0.326 |
| Unemployed | 0.408 | 0.176 | 0.422 | 0.173 |
| **Father’s employment** | | | | |
| Full-time employed | 0.924 | 0.903 | 0.905 | 0.903 |
| Part-time employed | 0.035 | 0.045 | 0.042 | 0.041 |
| Unemployed | 0.041 | 0.052 | 0.053 | 0.056 |
| **Control Variables** | | | | |
| ***Age in years*** | 0.366 | 0.667 | 0.231 | 0.412 |
| Male | 0.508 | 0.508 | 0.506 | 0.506 |
| Female | 0.492 | 0.492 | 0.494 | 0.492 |
| ***Areas of residence*** | | | | |
| Accessible city areas | 0.957 | 0.957 | 0.956 | 0.956 |
| Not accessible regional areas | 0.043 | 0.043 | 0.042 | 0.04 |
