# Supplementary material for: Socioeconomic inequalities in childhood and adolescent obesity in Australia: The role of behavioral and biological factors
Source: PLoS One. 2025 Apr 16;20(4):e0321861. doi: 10.1371/journal.pone.0321861 (PMC12002548; doi:10.1371/journal.pone.0321861)
Supplement: S4 Appendix — (DOCX) [file pone.0321861.s004.docx]

**Mediation analysis of independent variable on the outcome variable (Obesity)**

| **Components** | **Consumption of fatty foods and BMI** | **Consumption of fruit and Vegetables and BMI** | **Drinking sugary beverages and BMI** | **Activities during free time and BMI** | **Outdoor activities and BMI** |
| --- | --- | --- | --- | --- | --- |
|  | Coefficient | Coefficient | Coefficient | Coefficient | Coefficient |
| ACME | 0.000 | -0.000246  * | 0.0001 | 0.00047 | 0.0005  *** |
| ADE | 0.117  *** | 0.117239  *** | 0.117  *** | 0.117  *** | 0.117  *** |
| Total Effect | 0.117  *** | 0.116993  *** | 0.117  *** | 0.117  *** | 0.117  *** |
| Proportion Mediated | -0.001 | -0.0021  * | 0.0013 | 0.004 | 0.0046  *** |

**Note: ACME: Average Causal Mediation Effect, ADE: Average Direct Effect, Total Effect**: **Prop. Mediated: Proportion Mediated**

**Mediation analysis on Consumption of fatty foods and Obesity**

In summary, the direct and total effects of the independent variable on the outcome are significant, while the mediation effect is negligible and not statistically significant.

**Mediation analysis on Consumption of fruit and Vegetables and Obesity**

the direct and total effects of the independent variable on the outcome are strong and significant, the mediation effects are small and negative, indicating that the mediator slightly reduces the overall effect. Both the direct and mediated effects are statistically significant, suggesting they are unlikely to be due to random chance.

**Mediation analysis on Drinking sugary beverages and Obesity**

The direct and total effects of the independent variable on the outcome are strong and statistically significant, the effects mediated by the mediator are minimal and not statistically significant.

**Mediation analysis on Activities during free time and Obesity**

In simple terms, while the direct and total effects of the independent variable on the outcome are significant and strong, the mediated effects—both the ACME and the proportion mediated—are very small and not statistically significant.

**Mediation analysis on Outdoor activities and Obesity**

Both the direct effects and the effects through a mediator of the independent variable on the outcome are significant. The mediated effects, though small and contribute to how the independent variable impacts the outcome
