# Supplementary material for: Socioeconomic inequalities in childhood and adolescent obesity in Australia: The role of behavioral and biological factors
Source: PLoS One. 2025 Apr 16;20(4):e0321861. doi: 10.1371/journal.pone.0321861 (PMC12002548; doi:10.1371/journal.pone.0321861)
Supplement: Attachment — Submitted filename: Evaluation_Report_PONE-D-24-34935.pdf [file pone.0321861.s005.pdf]

# Evaluation Report

**(PONE-D-24-34935)**

**Socioeconomic Inequalities in Childhood and Adolescent Obesity in Australia: The role of behavioral and biological factors**

This article provides an in-depth examination of how biological and behavioral factors contribute to obesity disparities across socioeconomic groups in Australian children and adolescents. The submitted work unfolds that the childhood and adolescent obesity is a major public health issue shaped by biological and behavioral factors. This study explored how these factors contribute to socioeconomic disparities in obesity among Australian children and adolescents. Using data from the Longitudinal Study of Australian Children, the analysis revealed that household income (49.17%), biological factors (28.96%), and behavioral factors (10.36%) significantly influence these disparities. The findings underscore the need for targeted public health strategies to support low-income families and reduce obesity-related inequalities.

The use of longitudinal data from the Longitudinal Study of Australian Children (LSAC) adds significant strength, allowing for a nuanced understanding of obesity determinants over time. The application of Concentration Index and Decomposition Index analyses provides a clear quantification of contributions to socioeconomic inequalities. Moreover, the integration of both biological (e.g., parental BMI) and behavioral (e.g., dietary habits and physical activity) factors offers a holistic perspective.

Thus, this article makes a significant contribution to understanding the interplay between socioeconomic factors and childhood obesity. However, following are two minor observations need to be addressed before its acceptance for publications:

1. While behavioral factors are included, the study could further explore external environmental influences, such as urban design and access to recreational facilities, which might shape physical activity levels.
2. Despite the longitudinal design, some interpretations appear static, potentially overlooking temporal shifts in socioeconomic conditions.

(End of evaluation)
