# Supplementary material for: Socioeconomic inequalities in childhood and adolescent obesity in Australia: The role of behavioral and biological factors
Source: PLoS One. 2025 Apr 16;20(4):e0321861. doi: 10.1371/journal.pone.0321861 (PMC12002548; doi:10.1371/journal.pone.0321861)
Supplement: Attachment — Submitted filename: Evaluation_Report_PONE-D-24-34935R1.pdf [file pone.0321861.s007.pdf]

Evaluation Report

(PONE-D-24-34935R1)

Socioeconomic Inequalities in Childhood and Adolescent Obesity in Australia: The role of behavioral and biological factors

The minor observations have been addressed and the article is now suitable for acceptance.

(End of evaluation)
