# Supplementary material for: Socioeconomic inequalities in childhood and adolescent obesity in Australia: The role of behavioral and biological factors
Source: PLoS One. 2025 Apr 16;20(4):e0321861. doi: 10.1371/journal.pone.0321861 (PMC12002548; doi:10.1371/journal.pone.0321861)
Supplement: Attachment — Submitted filename: Editor response.docx [file pone.0321861.s006.docx]

1.Please upload a copy of the original ethics approval letter, as required by question 1 of the human subject research checklist. If the original letter is not in English, please also provide an English-translated version in the supporting file. Please note that if this documentation is not included when your manuscript is resubmitted, it may be rejected.

**Our response**

Thank you for your inquiries, this study utilized secondary data from the Longitudinal Study of Australian Children (LSAC). As such, ethical approval was not required for this particular analysis. However, to access the LSAC data, the authors or researchers must obtain permission from the LSAC. Additionally, the ethical clearance for the LSAC data, as granted by the Ethics Committee of the Australian Institute of Family Studies, is detailed in the Methods section of our study (please see Lines 236 to 242).

2). Before we can proceed, please report in the Methods section the dates when data were accessed for research purposes. Please note that if this information is not included when your manuscript is resubmitted, it may be rejected

**Our response**

Thank you for your feedback. The authors accessed the dataset titled “Growing Up in Australia: Longitudinal Study of Australian Children (LSAC) Release 9.0 C2 (Waves 1-9C)” on April 28, 2023 (please see Lines 236 to 242).
